# Assessing the Impact of a Health Education Anti-Smoking Program for Students: A Follow-Up Investigation

**DOI:** 10.3390/children11040387

**Published:** 2024-03-23

**Authors:** Maria Angeli, Mary Hassandra, Charalampos Krommidas, Ioannis Morres, Yannis Theodorakis

**Affiliations:** Department of Physical Education and Sport Science, University of Thessaly, 42100 Trikala, Greece; marangeli@uth.gr (M.A.); mxasad@uth.gr (M.H.); hkrom@uth.gr (C.K.); iomorres@pe.uth.gr (I.M.)

**Keywords:** adolescents, smoking, education, intervention, prevention, theory of planned behavior, life skills

## Abstract

In this follow-up study, we aimed to assess the effectiveness of the “*I do not smoke, I exercise*” anti-smoking preventive health education program. The program was based on the theory of planned behavior supplemented with life skills teaching and targeted at high school students. The intervention comprised ten one-hour online sessions, administered by physical education instructors. The study cohort comprised 222 students (109 boys, 113 girls) from 11 secondary schools, with an average age of 16.42 ± 1.36 years. Data collection involved pre- and post-intervention self-assessment questionnaires. The examined variables included attitudes towards smoking, intention to smoke, subjective norm, perceived behavioral control (PBC), knowledge about smoking, smoking behavior, exercise behavior, attitudes toward the program’s implementation, and satisfaction with the program. A separate paired samples *t*-test revealed a significant improvement in students’ knowledge about smoking (*t*_217_ = −5.605, *p* < 0.001, d = 0.38) and perceived behavioral control (*t*_220_ = −2.166, *p* < 0.05, d = 0.15) following the intervention. However, no significant changes were observed in the remaining variables. In addition, students’ overall satisfaction with the implementation of the present health education program was high (*M* = 5.72 ± 1.39). These findings suggest that the health education smoking prevention intervention incorporates techniques and strategies that influence the perceived behavioral control variable, emphasizing students’ strong interest in educationally theorized programs integrating technology into their design. Future studies should consider further examination of tobacco control strategies within the high school context.

## 1. Introduction

Tobacco contributed to a substantial portion of the worldwide disease burden in 2019, accounting for 15.4% of all deaths (approximately 8.71 million) [1], primarily attributed to non-communicable diseases associated with smoking [2]. The existing literature provides substantial evidence that cigarette smoking is associated with a broad spectrum of diseases [3], notably lung cancer and other types of cancer [4], chronic obstructive pulmonary disease [4,5], and types of cardiovascular diseases [6]. Given that smoking leads to the death of approximately half of its consumers [7], preventing the initiation of smoking and implementing targeted interventions implies substantial long-term health benefits [8].

Healthy behaviors established in early life tend to persist into adulthood [9]. Smoking behavior typically takes root during adolescence, with the majority of adolescents trying their first cigarette or becoming addicted before reaching the age of 18 [10,11]. For example, over 80 percent of adult heavy smokers were heavy smokers during adolescence [12]. Thus, interventions aimed at preventing unhealthy behaviors among youth, particularly smoking, have strategically focused on the school environment [13]. There is empirical evidence confirming the efficacy of integrated school health approaches in reducing rates of various adolescents’ health behaviors, including smoking [14,15,16]. As illustrated by a comprehensive examination of systematic reviews conducted by Das et al. [17], it was indicated that school-based preventive initiatives and intensive family-centered interventions have shown effectiveness in reducing smoking behaviors.

In recent years, there has been a substantial surge in internet accessibility and computer utilization, notably among the younger demographic, wherein a significant portion of their time is allocated to computer-based activities [18]. Web-based health education programs have shown a positive impact on children’s and adolescents’ health behaviors [19,20]. When considering smoking prevention initiatives, the results are promising, indicating their effectiveness in preventing smoking initiation, particularly within the demographic subset aged 14–16 years [21,22].

Ajzen’s [23] theory of planned behavior (TPB) attempts to elucidate the rationale behind human behavior by describing individuals’ actions in a systematic framework. The TPB postulates that the intention to perform a behavior, that is, an individual’s motivation to act, serves as the actual precedence for behavior. The intention to perform a behavior is influenced by three elements, attitudes, perceived behavioral control, and subjective norms. An individual’s attitude towards the behavior, that is, the evaluation of the behavior (positive or negative), the perceived impact of influential individuals on behavior and their adoption of it, subjective norm, and the perceived capability to engage in the behavior, which stands for PBC, which also impacts directly the behavior [23,24].

Ajzen [24] emphasizes the importance of incorporating a theory in interventions targeting smoking behavior, as the belief in one’s capacity to influence behavior significantly impacts both intentions and actual actions. Steinmetz et al. [25] found that interventions based on the TPB had a notable positive impact on changing various health behaviors and related factors according to their meta-analysis. Recent studies have effectively used the TPB to predict the link between smoking behavior and TPB’s variables [26,27]. Moreover, it has been effectively used to design, implement, and evaluate theory-based, behavior-change antismoking interventions [28,29].

Lareyre et al. [30], after conducting a systematic review of TPB-based interventions for smoking behavior, found that the percentage of studies reporting a significant impact on smoking behavior, intention, attitude, subjective norm, or perceived behavioral control ranged from 42% to 50%. For example, results of studies applying health education behavior change interventions utilizing the TPB, intending to prevent smoking among adolescents, indicate significant effects on the TPB constructs post-intervention [29,31,32,33].

Health promotion interventions have the potential to impact various dimensions of the self, including cognitive, behavioral, and motivational aspects, on an individual level [34]. Health education was initially defined as the capacity to comprehend, assess, and utilize health information to make informed decisions about one’s health [35]. Systematic reviews highlight the effectiveness of school-based smoking prevention programs for children and adolescents, emphasizing a combination of social competence and social influences curricula in preventing smoking initiation [13,36].

Long-term research has demonstrated the positive effects of life skills teaching on various substance use behaviors, including smoking [37]. In what manner do life skills contribute to the well-being of adolescents? They do so by furnishing them with the requisite knowledge, positive attitudes, and values necessary for a healthy future [38]. Skills-based health education involves specifying knowledge, attitudes, and life skills to guide young individuals in adopting targeted behaviors and meeting outlined objectives, encompassing psychosocial competencies and interpersonal abilities crucial for effective decision-making, problem-solving, critical thinking, communication, relationship-building, empathy, and productive life management [39]. The incorporation of life skills into smoking reduction programs, whether delivered through cognitive behavior, or life skills modalities and implemented within a school-community framework, has demonstrated the potential to enhance adolescent smoking reduction rates [40,41]. As an illustration, Velasco et al. [42] conducted a drug abuse prevention program for Italian adolescents, employing a life-skills-based approach. Encouragingly, this intervention demonstrated positive effects, not only in terms of reducing substance use behavior, and associated variables, but also in bolstering overarching protective factors such as normative beliefs, life skills, and various psychosocial outcomes. Zhao et al. [33] implemented and evaluated a brief antismoking intervention based on extended TPB and assisted by life skills, that targeted high school students. Improved attitudes towards smoking and PBC were observed, but no significant improvements in intentions, subjective norms, and smoking behavior were observed. The authors suggested a focus on sessions that target attitudes.

Substantial evidence supports the benefits of exercise as an effective option for alleviating tobacco symptoms of withdrawal and urges [43,44]. Experimental research has examined the acute and long-term effects of exercise on smoking behavior, offering valuable information for developing antismoking interventions [45,46,47]. The literature findings demonstrate that adolescents can effectively improve multiple health behaviors, with positive changes in both activity levels and smoking cessation [48,49]. On top of these, interventions grounded in the TPB have demonstrated effectiveness in promoting physical activity behavior when compared to interventions based on other theories [50,51].

In sum, the above evidence encourages the development and implementation of a theory-based, school, health education program, targeting smoking prevention among adolescents. This article represents a follow-up study building upon prior research conducted by Kolovelonis et al. [29]. This follow-up study examines the effectiveness of the “*I do not smoke, I exercise*” web-based program, targeting a healthier lifestyle and smoking prevention among secondary students. The program prioritizes providing children with competencies to make healthy decisions and prevent the onset of smoking behavior. The study evaluates an extended version by Kolovelonis et al. [29], featuring ten tailored sessions for adolescents, with the hypothesis of positive effects on examined variables.

## 2. Materials and Methods

### 2.1. Participants

Eleven physical education instructors voluntarily implemented the program, with both teachers and students participating in the study of their own accord. In total, 11 secondary schools and 222 students (109 boys and 113 girls; Mage: 16.42 ± 1.36 years) voluntarily participated in the school-based intervention program “I do not smoke, I exercise”. The eligibility criteria encompassed secondary students aged 13–17 consenting to participation with parental approval. Conversely, exclusion criteria involved non-participation in the program and questionnaire incompleteness. The data collection period was between 21 February 2018 and 4 January 2021.

### 2.2. Instruments

The assessment scales utilized in this study were grounded in the TPB model, encompassing measures of attitudes towards smoking, intention to smoke, subjective norm, and perceived behavioral control over smoking behavior, as outlined by Ajzen [24,52]. These scales, previously employed in research by Kolovelonis et al. [29], were also translated into Greek for the purposes of this study. Exercise behavior, attitudes toward the program, and satisfaction with the program’s content were also assessed. Furthermore, similar assessment tools have been employed to gauge the effectiveness and suitability of health education programs targeting behaviors like nutrition [45].

*Attitudes towards smoking*. Attitudes towards smoking behavior were evaluated using a set of five questions “*For me smoking is…*”. The responses were recorded on a 7-point bipolar adjective scale, encompassing dimensions such as good–bad, silly–clever, healthy–unhealthy, pleasant–unpleasant, and useful–unuseful.

*Intention to smoke*. The assessment of intention to smoke involved the inclusion of two specific items (e.g., “*I plan to smoke in the coming months*” and “*I will try to smoke in the coming months*”). Participants rated their responses on a 7-point Likert scale, ranging from 1 (signifying strong disagreement) to 7 (signifying strong agreement).

*Subjective norm*. Subjective norm assessment involved the use of two items: “*People important in my life believe that I will smoke in the next months*” and “*Great people in my life will like me to smoke in the next months*”. Participants indicated their responses on a 7-point Likert scale, ranging from 1 (indicating strong disagreement) to 7 (indicating strong agreement).

*Perceived behavioral control (PBC)*. Participants’ perceived competencies in exerting control over their smoking behavior were evaluated using two items: “*It’s entirely up to me whether I will smoke in the coming months*” and “*It is my responsibility if I smoke in the coming months*”. Responses were recorded on a 7-point Likert scale, ranging from 1 (strongly agree) to 7 (strongly disagree).

*Attitudes towards the program implementation*. Participants were asked to provide their responses within the context of the prompt “*For me, the program is…*” using a 7-point Likert scale, which included bipolar word pairs such as good–bad, pleasant–unpleasant, attractive–unattractive, and useful–useless.

*Satisfaction with the program*. Participants’ satisfaction with the program was assessed post-intervention, using six items (“*Using this program* via *computer and the internet was easy*”, “*Using the computer and the internet through this program was easy*”, “*I prefer to learn about such issues* via *the computer and the internet, rather than from a book*”, “*I am satisfied with the texts in this program*”, “*I am satisfied with the design of this program*”, “*I am satisfied with the images I saw in the program*”). Responses were recorded on a 7-point Likert scale, ranging from 1 (indicating complete disagreement) to 7 (indicating complete agreement).

*Knowledge about smoking*. A 14-item questionnaire was developed to assess participants’ knowledge regarding smoking, specifically focusing on the health consequences associated with smoking behavior. The questionnaire included the following items: “*Smoking brings difficulty breathing when exercising*”, “*Smoking hardens and destroys arteries*”, “*Smoking causes cancer*”, “*Smoking causes bronchitis*”, “*People who inhale more smoke than others are at greater risk of cancer, heart disease and stroke*”, “*Children who inhale more smoke than others are more likely to get colds, infections, asthma and respiratory problems*”, “*Children born to parents who smoke are more likely to develop asthma, infections and other diseases*”, “*Second-hand smoke from cigars is more dangerous than second-hand smoke from cigarettes*”, “*The percentage of Greek men and women who smoke is among the highest in Europe*”, “*The percentage of Greek men and women exercising is among the lowest in Europe*”, “*Many smokers start smoking before the age of 14, with 90% of smokers starting before the age of 19*”, “*Tobacco companies target young people, try to make smoking look attractive, fun, or sexy*”, “*Many young people smoke because they think they will grow up faster*”, and “*Many young people smoke because it makes them feel accepted by their friends*”. The questionnaire has been modified to the program’s sessions. It covered the dangers of smoke inhalation, nicotine addiction, statistics regarding the prevalence of smoking in Greece compared to Europe, and causes that lead youth to start smoking. Participants responded to the questions using a 3-point scale (0 = no, 1 = I am not sure, 2 = yes), with scores from 0 to 28.

*Exercise behavior* was assessed with the question: “*In your free time, how often do you engage in physical activity that induces sweating or heavy breathing?*” on a 7-point scale (0 = *never*, 6 = *every day*) [53].

*Smoking behavior* was evaluated using an assortment of five yes-or-no questions based on a questionnaire used in previous research [54]. These questions gradually examined a participant’s smoking history, starting with “*Have you ever tried smoking, even just 1 or 2 puffs?*”, “*Have you ever smoked 1 or more cigarettes every day for a whole month (30 days)?*”, “*Have you smoked even one day in the past month (30 days)?*”, “*Have you smoked on 20 or more days in the last month?*” and concluding with “*Do you smoke more than 10 cigarettes per day?*”.

### 2.3. Procedures

All participating students and their parents/guardians provided their consent to partake in the study. The program’s materials and assessments were administered through the website http://research.pe.uth.gr/health/ (assessed on 10 July 2022). Teachers who expressed interest in implementing the program completed a participation form and were subsequently provided access to the program’s materials. Students utilized the usernames provided for pre-intervention questionnaires and reused them for post-intervention assessments. Teachers who successfully completed all sessions and encouraged student participation were awarded a certificate of attendance, in addition to receiving the evaluation results. The program offered downloadable materials featuring instructions, relevant sources, presentations, activities, games, teaching strategies, project work topics, pertinent websites, and more. It comprised ten one-hour sessions, as outlined in Table 1.

### 2.4. Data Analysis

Descriptive statistics (means, standards deviations) and reliability index (Cronbach’s *α*) were calculated for both measurements. Then, separate paired samples *t*-tests with Cohen’s *d* effect sizes were calculated in order to examine possible differences in the dependent variables (attitudes toward smoking, intention to smoke, subjective norms, PBC, attitudes toward the program’s implementation, smoking knowledge, smoking behavior and exercise behavior) between pre- and post-intervention. All statistical analyses were conducted with IBM SPSS v 26.0. The *p*-value was set at 0.05.

## 3. Results

Results from the paired samples *t*-tests showed significant differences in students’ PBCs (t_220_ = −2.166, *p* < 0.05, d = 0.15), and knowledge about smoking (t_217_ = −5.605, *p* < 0.001, d = 0.38) between pre and post-intervention measures. Students reported higher scores on PBC and knowledge about smoking after the implementation of the “*I do not smoke, I exercise*” health education program compared to their pre-intervention measure (Table 2). Students also reported that they were very satisfied with the implementation of the “*I do not smoke, I exercise*” health education program. In contrast, there were no significant differences in students’ attitudes towards smoking (t_221_ = 1.418, *p* = 0.158), intention to smoke (t_221_ = 0.534, *p* = 0.594), subjective norm (t_221_ = 0.193, *p* = 0.847), attitudes toward the program’s implementation (t_221_ = −0.975, *p* = 0.331), smoking behavior (t_220_ = −0.907, *p* = 0.365), and exercise behavior (t_221_ = −1.232, *p* = 0.219).

## 4. Discussion

The current study aimed to assess the effectiveness of a school-based health education program focused on preventing smoking among adolescents. Overall, the implementation of the program was deemed satisfactory. The findings highlighted that the most substantial impact of the program, as indicated by effect sizes, was the enhancement of students’ knowledge of smoking behavior and the PBC variable. In accordance with previous research findings, these results demonstrate that health education programs exert a notable influence on improving students’ knowledge [28,29,45]. Certainly, targeted education has been shown to enhance individual awareness and proficiency [55]. There is some evidence that improving health literacy through health education can be advantageous in preventing smoking, aiding in smoking cessation, and preventing smoking relapse, along with addressing associated behaviors and issues [56]. However, a mere increase in knowledge does not necessarily translate to behavior change [57], although Sorensen et al. [58] have found that an increase in knowledge aids in fostering positive attitudes towards abstaining from smoking.

The program also demonstrated a notable impact on the PBC variable, a finding consistent with previous studies [29,33,59]. This suggests that the incorporation of life skills and the use of behavior change techniques, such as behavioral practice and rehearsal, enhanced students’ PBC and self-efficacy [60]. Relevant research demonstrated the significance of the PBC variable on students smoking behavior [61,62].

Consistent with Kolovelonis et al.’s [29] findings, no improvements were observed in students’ attitudes towards smoking, intention to smoke, and subjective norms post-intervention. This might be attributed to the existing weak pre-intervention attitudes toward smoking. Applying the intervention to a targeted group, consisting rigorously of smokers or non-smokers, could lead to more explicit inferences. Concerning the subjective norms variable, Zhao et al. [63] found that the subjective norm emerged as the most resistant construct to antismoking intervention efforts. Also, a previous study (Zhao et al. [33]) suggested that displayed a limited association with the intention variable. Home environment, modeling, and mass media have a substantial effect on forming youth’s smoking behavior [64,65]. Thus, future studies should consider influencing this context, targeting students’ peers and families.

Smoking behavior and exercise behavior remained unaffected post-intervention. Results are in line with previous studies [29,33] which indicated that their intervention failed to change smoking behavior. Davey et al. [66] noted that subjective norm exerts a potent influence on intention, and the challenge of altering this norm may be a factor contributing to the modest changes observed in smoking behavior. An alternative interpretation could be that students initially reported low levels of smoking, which may have posed a challenge in detecting significant changes in the post-test results. Nevertheless, it is noteworthy that a recent systematic review by Lareyre et al. [30] revealed that none of the studies focusing on smoking prevention provided results on smoking behavior. Furthermore, exercise behavior showed no significant changes in the post-test, aligning with the earlier study by Kolovelonis et al. [29]. This limited impact could be attributed to the high levels of exercise behavior reported at the baseline.

In summary, students overwhelmingly welcomed the program, signifying a substantial influence on their satisfaction levels. This aligns with findings from comparable studies, highlighting students’ keen interest in educationally theorized programs. Moreover, research outcomes [28,29,45] indicate that integrating technology into health education program delivery serves as an effective method for augmenting the quality and effectiveness of these endeavors.

Regarding the limitations of the study, the absence of a control group should be noted. Furthermore, these differences among aspects of TPB may be attributed to sample differences (different age groups and school contexts). It appears that interventions aimed at the adolescent population should be addressed distinctly, as these findings pertain specifically to this demographic. Future studies should contemplate the development of age-specific interventions. The implementation of the program in different ages and groups may give more efficient results. The employment of self-assessment questionnaires and the implementation fidelity which is always an issue in this kind of online material training and intervention programs [67], represent two potential constraints that could exert an influence on the outcomes. Future school education programs should consider the participation of peers and family and assess the impact of life skills on students employed in the intervention.

## 5. Conclusions

In general, this intervention demonstrated limited effectiveness in positively influencing participants’ smoking behavior and its immediate cognitive precursors, such as attitudes and intentions. Favorable outcomes were observed only in participants’ knowledge, perceived behavioral control (PBC), and overall program satisfaction. These results carry significant implications for smoking prevention initiatives within the context of Greece. This study underscores the imperative for additional scrutiny of tobacco control strategies within the high school settings of Greece.

## Figures and Tables

**Table 1:** Description of the “I do not smoke, I exercise” web-based program.

Sessions	Theoretical Construct	BCT	Contents	Activities
1st	Attitudes Education	Shaping knowledge Natural consequences	Initial Evaluation—General knowledge about smoking	Create an antismoking message—Knowledge quiz: “What do we know about smoking?”
2nd	Education Attitudes Knowledge	Shaping knowledge Salience of consequences	Presentation: Research data about smoking—harmful effects of smoking on our body	Discuss; “what are the ingredients of a cigarette”—“Smoking causes…”—Passive smoking: “Is it dangerous?”—True or False?—Create your antismoking message—Homework in groups: I reread the information in the material and create a knowledge quiz”
3rd	Education Perceived behavioral control. Intention Life skills	Goal setting Monitoring of behavior Behavior substitution	Physical activity as an alternative to smoking behavior—Why exercise is important—what is aerobic exercise	Learn the benefits of exercise—presentation of research data- Discussion: “Does smoking affect my ability to exercise?”—Create your own positive and encouraging message about PA and smoking.
4th	Subjective norm Attitude Life skills	Behavioral experiments Health consequences Motivational interviewing	Effects of tobacco consumption—Smoking behavior and your body—What you need to know	Experimental activity: “Smoking bottle”—Presentation: “Smoking: effects on the body”—Discussion: “Why nicotine is addictive?”—Work team: “Interview family members, smokers & non-smokers”
5th	Education Perceived behavioral control. Attitudes Life skills	Information about social and environmental consequences Salience of consequences	Social effects of smoking—The negative effects of smoking compared to its enticing image	Facts: “Why do young people smoke?”—Worksheet: “Why do I have to imitate???”—Tobacco industry’s campaign to hide the hazards of smoking: Demystification of smoking advertisements
6th	Knowledge Life skills Attitudes	Behavioral rehearsal Shaping knowledge Comparative imagining of future outcomes	Practicing refusal skills—find ways to intelligently say NO to someone who offers you a cigarette—How an addiction can become more important to a person than family, friends, clubs, job, reputation	Brainstorming: “The BIG NO”—Teamwork: “Practice your big NO’s in possible scenarios”—Activity: “The red balloon”—create a poster with antismoking messages
7th	Perceived behavioral control Life skills	Action planning Problem-solving Commitment	Introduce a goal-setting technique—Introduction to problem-solving technique	“Setting my goals on smoking”—Brainstorming: “Barrier or Justification?”—Long-term vs. Short term consequences of smoking
8th	Knowledge Life skills Perceived behavioral control	Action planning Commitment	Practice goal-setting and problem-solving techniques—Use these techniques to set your activity goals	Self-assessment quiz: “How active are you?”—Set your goals to exercise daily—Physical activity pyramid
9th	Subjective norm Attitudes Life skills	Information about antecedents Provide healthy alternatives Comparative imagining of future outcomes	Transmit knowledge to others	Teamwork: “Develop a campaign about smoking and exercise”—Work in groups and create anti-smoking posters—“Share what you have learned with family and friends”
10th	Attitudes Subjective norm	Social comparison	Raising awareness for the harm smoking causes	Work in groups: “Writing Down Goals & Rewarding Yourself”–Organize an information day and inform your classmates about the consequences of smoking and the health benefits of exercise

**Table 2:** Descriptive statistics, reliability index and significant differences between pre- and post-intervention measures on the “I do not smoke, I exercise” health education program.

	Pre-Intervention			Post-Intervention		
Variables	α	M	SD	α	M	SD
Attitudes towards smoking	0.78	1.40	0.58	0.73	1.34	0.62
Intention to smoke	0.60	1.18	0.50	0.85	1.15	0.59
Subjective norm	0.59	1.33	0.77	0.84	1.32	0.88
PBC	0.48	4.51 *	2.07	0.86	4.92 *	2.40
Attitudes toward the program’s implementation	0.90	5.31	1.61	0.90	5.43	1.68
Knowledge about smoking	0.68	21.67 ***	3.74	0.80	23.45 ***	4.03
Smoking behavior	0.73	0.31	1.14	0.79	0.38	1.23
Exercise behavior	-	4.50	1.46	-	4.64	1.51
Satisfaction of the program	-	-	-	0.90	5.72	1.39

M: mean; SD: standard deviation; PBC: perceived behavioral control; α: Cronbach’s α reliability index; * *p* < 0.05; *** *p* < 0.001.

## Data Availability

The data presented in this study are available on request from the corresponding author. The data are not publicly available due to privacy and ethical restrictions.
